# Case report: Effectiveness of sirolimus in a *de novo* FAS mutation leading to autoimmune lymphoproliferative syndrome-FAS and elevated DNT/Treg ratio

**DOI:** 10.3389/fped.2022.868193

**Published:** 2022-07-28

**Authors:** Hao Gu, Zhenping Chen, Jie Ma, Jingyao Ma, Lingling Fu, Rui Zhang, Tianyou Wang, Runhui Wu

**Affiliations:** ^1^Hematology Center, Beijing Key Laboratory of Pediatric Hematology Oncology, National Key Discipline of Pediatrics, Key Laboratory of Major Diseases in Children, Ministry of Education, National Center for Children’s Health, Beijing Children’s Hospital, Capital Medical University, Beijing, China; ^2^Hematology Center, Hematologic Disease Laboratory, Beijing Key Laboratory of Pediatric Hematology Oncology, National Key Discipline of Pediatrics, Key Laboratory of Major Diseases in Children, Ministry of Education, National Center for Children’s Health, Beijing Children’s Hospital, Beijing Pediatric Research Institute, Capital Medical University, Beijing, China

**Keywords:** FAS (APO-1/CD95), ALPS (autoimmune lymphoproliferative syndrome), sirolimus (rapamycin), DNT, Treg - regulatory T-cell

## Abstract

**Background:**

The autoimmune lymphoproliferative syndrome (ALPS) is a rare disease characterized by defective function of the FAS death receptor, which results in chronic, non-malignant lymphoproliferation and autoimmunity accompanied by elevated numbers of double-negative (DN) T cells (T-cell receptor α/β + CD4–CD8–) and an increased risk of developing malignancies later in life.

**Case description:**

Here, we report a patient with a *de novo* FAS mutation with a severe phenotype of ALPS-FAS. The FAS gene identified as a novel spontaneous germline heterozygous missense mutation (c.857G > A, p.G286E) in exon 9, causing an amino acid exchange and difference in hydrogen bond formation. Consequently, the treatment with sirolimus was initiated. Subsequently, the patient’s clinical condition improved rapidly. Moreover, DNT ratio continuously decreased during sirolimus application.

**Conclusion:**

We described a novel germline FAS mutation (c.857G > A, p.G286E) associated with a severe clinical phenotype of ALPS-FAS. Sirolimus effectively improved the patient clinical manifestations with obvious reduction of the DNT ratio.

## Introduction

Apoptosis induced by the FAS-FAS ligand (FASL) pathway is vital in the regulation of the immune system. Under normal conditions, T-cell activation induces the expression of FASL, which can bind to FAS receptors on the same or nearby cells. The interaction of FAS and FASL results in the association of the adaptor protein FAS-associated death domain (FADD) and the recruitment of procaspases 8 and 10, which leads to the formation of a multimolecular signaling complex called death-inducing signaling complex (DISC) (1). DISC can propagate a signal leading to terminal caspase activation and apoptosis. This process is known as activation-induced cell death (AICD) (2). Extracellular region FAS mutations can induce low FAS expression due to nonsense-mediated RNA decay or protein instability, resulting in the defective death-inducing signaling complex formation and impaired apoptosis (3).

The autoimmune lymphoproliferative syndrome (ALPS) is an inherited disorder characterized by defective function of the FAS death receptor, which results in chronic, non-malignant lymphoproliferation and autoimmunity accompanied by the elevated numbers of double-negative (DN) T cells (T-cell receptor α/β^+^CD4^–^CD8^–^) and an increased risk of developing malignancies later in life (4). ALPS-FAS is most frequently caused by heterozygous somatic or germline mutations that generate mutant FAS proteins, often with defective death domains. Herein, we reported data from a patient with ALPS with a *de novo* FAS-exon 9 mutation, his parents, and healthy donors, including their clinical and laboratory findings.

## Case description

### Patient presentation

Herein, we present a case of 2-year-old boy with clinical and genetic characteristics of ALPS-FAS and TCRαβ + CD4/CD8 double-negative T cell (DNT) elevation. He showed unexplained large-scale splenomegaly and lymphadenopathy, along with multi-lineage autoimmune cytopenia at 2 years of age. The patient had elevated IgG:16.1(5.0–13.0)g/L and IgE:935(≤60)IU/ml [tested before intravenous immune globulin (IVIG)]. The patient was unresponsive to steroids, IVIG, platelet transfusions, and recombinant human platelet growth factors. The elevated proportion (>1.5% of total lymphocytes or >2.5% of CD3 + lymphocytes) of DNTs/CD3 + (6.8%) and chronic (>6 months), non-malignant, non-infectious splenomegaly made us consider the diagnosis of the required criteria of ALPS. However, he was positive for anti-platelet antibodies, anti-Epstein–Barr viruses, rubella viruses, cytomegalovirus, and herpes simplex virus immunoglobulin G. In addition, the analysis of markers for measles virus, parvovirus B19, schistosomiasis, and *Leishmania donovani* infection was negative. T lymphocytes (CD3 + CD19-) and T helper cells (CD3 + CD4 +) in peripheral blood samples were slightly increased. However, Treg/CD4 + ratio 2.5% (4.5–10%) was significantly decreased. Moreover, the patient displayed amplified proportions of cytokines IFN-γ:2.48 (0.00–2.10), IL-10:5.59 (1.20–4.55), and IL-6: 2.6 (0.00–2.05) pg/ml, especially IL-10, as a part of the secondary accessory diagnostic criteria for ALPS. Histological analysis of the bone marrow and the spleen did not show signs of hemophagocytosis with obviously splenomegaly. Because of the severe clinical manifestation, we tried the mTOR signal pathway inhibitor before the NGS genetic results, after which the platelet count was restored and the spleen retracted quickly. In addition, the patient’s familial history was negative.

### Genetic findings

**FIGURE 1:**
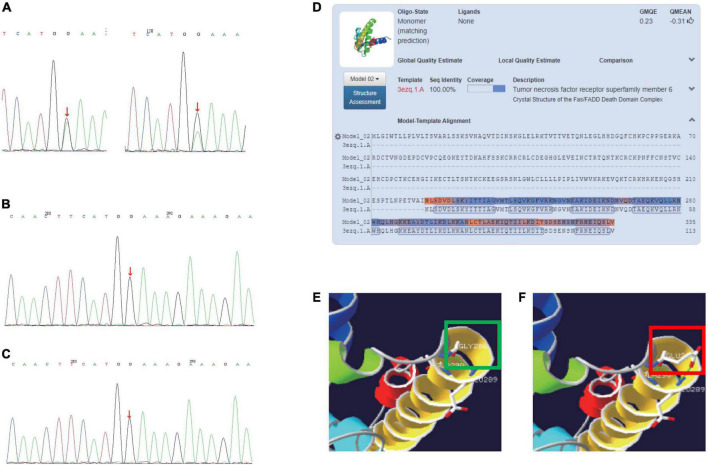
**(A)** Sanger sequencing of the FAS gene (c.857G > A, p.G286E) germlinemutation of patients (left: DNA of patient’s peripheral blood; right: DNA of patient’s nail). **(B)** Sanger sequencing of father’s FAS gene. **(C)** Sanger sequencing of mother’s FAS gene. **(D)** Crystal structure of the FAS/FADD death domain complex (3ezq.1.A). **(E)** A hydrogen bond is formed between amino acids 286G and 289E; a hydrogen bond is formed between amino acid 286G and 290A. **(F)** A total of two hydrogen bonds are formed between amino acids 286E and 289E; A hydrogen bond is formed between amino acid 286E and 290A. The green and red squares in panels **(E,F)** show the difference of hydrogen bond formation between wildtype and the mutation.

We sequenced the ALPS-related genes that may match the primary accessory criteria of ALPS (defective FAS-induced lymphocyte apoptosis somatic or germline pathogenic mutation in FAS, FASL, FADD, or CASP10) and the genes known to cause common variable immunodeficiency. We identified novel spontaneous germline heterozygous missense mutations on the FAS gene (c.857G > A, p.G286E) in exon 9 (Figures 1A–C), causing an amino acid exchange and difference of hydrogen bond formation (Figures 1D–F). Sanger sequencing verification of peripheral blood and nail confirmed the germline mutation. The identified variation, which has been previously observed, is the first disease-causing mutation detected in the intracellular domain of the FAS (neither in the 1,000 Genomes Project, the HapMap Project, Exome Variant Server data sets nor in the dbSNP database or the ExAC-Asian database). No mutations were detected by Sanger sequencing of the FASLG, CASP8, and CASP10 genes.

### Laboratory findings

**FIGURE 2:**
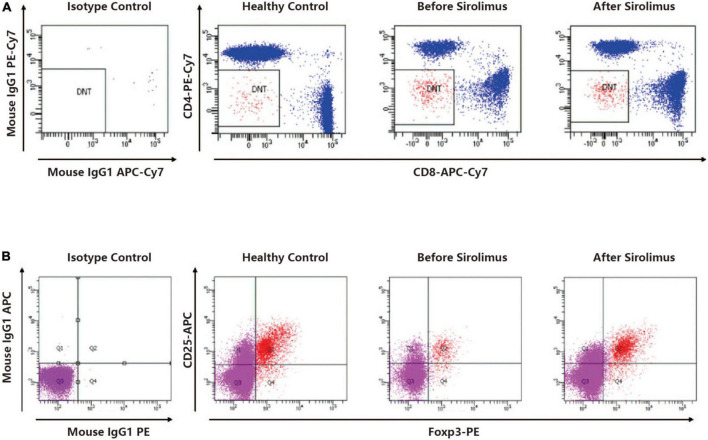
**(A)** A representative dot plot analysis of CD4-CD8-TCRαβ + cells gated in the CD3 + cell fraction showed elevated DNT ratio compared to a healthy control. The DNT ratio of the patient decreased after sirolimus treatment. **(B)** A representative dot polt analysis of CD25 + foxP3 + cells gated in the CD4 + cell fraction showed decreased Treg ratio compared to a healthy control. The Treg ratio of the patient increased after sirolimus treatment.

The patient displayed persisting T-cell lymphocytosis with an increased proportion of DNT, while Treg cells (CD3 + CD4 + CD25 + Foxp3 +) were reduced (compared to health control). After 4 weeks of rapamycin treatment, DNTs/CD3 + and DNTs/lymphocyte were decreased from 6.8 to 4.44% and 4.69 to 3.34%, respectively (Figure 2A). Treg increased to a normal level from 2.5 to 4.12% (Figure 2B).

### Clinical course

Medical history, clinical presentation [chronic (>6 months), non-malignant, non-infectious lymphadenopathy and splenomegaly], and laboratory test (elevated DNT ≥ 2.5% of CD3 + lymphocytes) results led to the required criterion of ALPS. In addition, the autoimmune cytopenias (hemolytic anemia, thrombocytopenia, or neutropenia) and elevated immunoglobulin G levels of this patient also met one of the secondary accessory ALPS diagnostic criteria.

**FIGURE 3:**
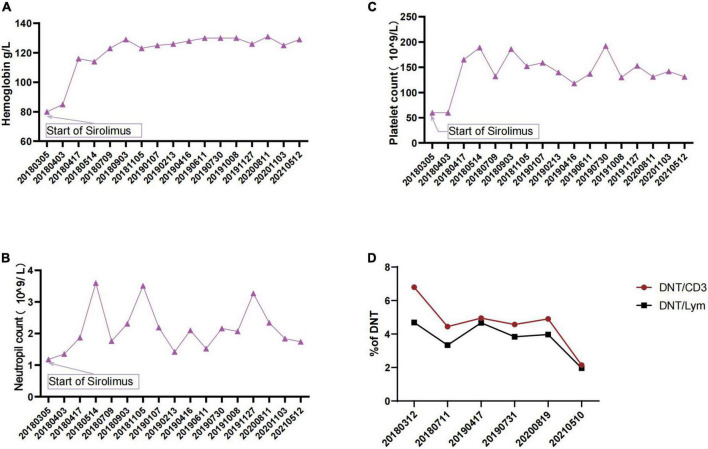
**(A)** Changes in patient’s hemoglobulin during sirolimus therapy. **(B)** Changes in patient’s neutrophil count during sirolimus therapy. **(C)** Changes in patient’s platelet count during sirolimus therapy. **(D)** Changes in DNTs during sirolimus therapy.

Consequently, the sirolimus treatment (1.5 mg/m^2^, blood concentration 4.74–11.05 ng/L) was initiated with the probable diagnosis (both required criterion and one secondary accessory criterion) of ALPS. Subsequently, the patient’s platelets and hemoglobulin levels improved rapidly (Figures 3A–C). The spleen length reduced from 7.5 to 1.3 cm. When the genetic findings came back, we gave a definitive diagnosis of ALPS-FAS (meet clinical required criteria for ALPS plus germline FAS mutation). Moreover, the DNT ratio continuously decreased during sirolimus application (Figure 3D).

## Discussion

The role of FAS in maintaining immune tolerance to prevent autoimmunity was clarified by the study of mice with the deficiency in the FAS antigen gene (5). These mice were discovered to have massive DNT cell proliferation, which indicates an important role for FAS antigen in the negative selection of autoreactive T cells in the thymus. FAS mutations can appear in a heterozygous and homozygous state. The first one has differencing clinical penetrance, whereas homozygous mutations have been associated with early onset and severe clinical manifestations (6). In this study, we reported a single case of a patient with typical ALPS caused by a heterozygous mutation in the FAS gene’s intracellular death domain, a member of the TNF/TNFR superfamily that contains nine exons spanning 26 kb on chromosome 10q24.1 (7). The first 5 exons encode the extracellular portion containing 3 cysteine-rich domains that control receptor trimerization and FASL binding, which could induce the proliferation of murine CD4 + T cells. The frequency of missense mutations in exon 9 is rare (8). To determine whether the mutations would impair protein function, we predicted the score of PolyPhen2 and SIFT (0.822 and 0.016, respectively). The frequency of EXAC-Asian (c.857G > A, p.G286E) was less than 0.00001. To predict the effect of the mutation on protein structure, we used a crystal structure of the FAS/FADD death domain complex (3ezq.1.A). Multiple counts of hydrogen bond difference between amino acids 286G, 286E, and 290A were discovered.

The data of a comprehensive report by the National Institute of Allergy and Infectious Diseases (NIAID) illuminated that FAS mutations have a clinical penetrance of <60%, and elevated serum vitamin B12 is a reliable and accurate biomarker of ALPS-FAS (9). The data also suggest that FAS mutations are not sufficient to cause clinical ALPS. The precise mechanism underlying disease pathogenesis remains unclear but may be related to the development of DNTs, which are significantly elevated in ALPS-FAS, especially ALPS-FASLG (10).

The differential diagnosis of inborn errors of immunity associated with lymphoproliferation is extremely complex. Interestingly, in the activated PI3Kδ syndrome [APDS; also known as p110δ-activating mutation causing senescent T cells, lymphadenopathy, and immunodeficiency (PASLI)], ALPS, EBV-related lymphoproliferative disorders, and regulatory T-cell disorders, lymphadenopathy is one of the leading signs of the entire clinical picture (11). In the described patients, the association between lymphoproliferation and immune cytopenia is the first and fundamental step of the diagnostic process (12). Also, screening for EBV infection and for other immunodeficiency-associated features is of clinical relevance. Before the analysis of DNTs, it is reasonable to state that ALPS or ALPS-related disorders have to be suspected in patients with unexplained splenomegaly or lymphadenopathy, especially when there is an association with autoimmune cytopenia, positive familial history, and elevated B12 levels. This could help in identifying the patients to be tested for other specific ALPS-associated markers. In patients with lymphadenopathy and/or splenomegaly with elevated DNT, ALPS should be suspected. Genetics and biomarkers can confirm this. All the patients with definite and suspected ALPS had elevated DNT when the test was performed. However, 51.5% of patients with unlikely ALPS diagnosis also showed abnormal DNT levels. Several types of immunodeficiencies, such as X-linked immunodeficiency with magnesium defect, Epstein–Barr virus infection and neoplasia (XMEN), autoimmune disorders, such as juvenile systemic lupus erythematosus, mixed connective tissue disease (MCD), and severe infections, can also cause elevated DNT (13). The combination of elevated DNT and an abnormal *in vitro* apoptosis functional test was the most useful to identify all types of patients with ALPS; the combination of the abnormal *in vitro* apoptosis functional test and elevated sFASL was a predictive marker for ALPS-FAS group identification. The combination of elevated sFASL and an abnormal apoptosis function was the most valuable prognosticator for patients with FAS mutations (13). The ALPS suspicion was facilitated by the significant polarization of the DNT/Treg axis in this patient. It has been reported that the acquisition of a somatic FAS mutation, which is typically enriched in DNT cells, can precipitate the development of full-blown ALPS (14). The inhibition of Treg may be due to the upregulation of IL-10 caused by DNT accumulation (15). Further investigation on DNT by CyTOF (Cytometry + Time Of Flight) elucidated that DNTs in patients with ALPS-FAS showed a distinct CD38 + CD45RA + population. Functionally, FAS-controlled T cells represent highly proliferative, non-cytotoxic T cells with an IL-10 cytokine bias. Mechanistically, the regulation of this physiological population is mediated by FAS and CTLA4 signaling, and its survival is enhanced by mTOR and STAT3 signals. Genetic alterations in these pathways result in the expansion of FAS-controlled T cells, which can cause significant lymphoproliferative disease (16).

The management of ALPS focuses on the treatment of disease manifestations and complications. The only known cure for ALPS is HSCT. However, because of the risks associated with HSCT, it is often performed only in those with very severe clinical phenotypes who are refractory to immune suppression (17). First-line treatment of ALPS often includes high-dose intravenous corticosteroids and intravenous immunoglobulin (IVIgG). Many patients often respond well to corticosteroids (1–2 mg/kg). Splenectomy and rituximab are commonly employed for autoimmune disease but are relatively contraindicated in patients with ALPS, especially in light of other more effective common therapies (18). The two most commonly used immunomodulatory drugs for ALPS are MMF and sirolimus. If lymphoproliferation is the predominant feature, more evidence supporting the use of sirolimus given its safety, tolerability, and most importantly efficacy are emerging (19). This is especially true in regard to patients with ALPS-FAS and therefore may not be applicable to all patients with ALPS. In spite of measured improvements in autoimmune disease, MMF has not been observed to cause lymphocyte death or has any effect on lymphoproliferative disease or depletion of DNTs. MMF that should be used for patients has intolerance to sirolimus (20). In this patient, we treated it with sirolimus. With the improvement of clinical manifestations, DNT also showed a significant decrease, though still in the ALPS criteria range. It is uncertain whether sirolimus affects expansion or induces apoptosis of DNT cells or both, but its therapeutic efficacy suggests specific signaling requirements of mTOR in DNT cells. Furthermore, various studies suggested that sirolimus promotes generation, expansion, and functionality of Tregs (21). However, the role of sirolimus in its contribution to modulating Tregs and their role in self-tolerance remain unclear.

This case highlights the importance of studying ALPS-FAS within the context of interconnected immune dysregulation disorders that have been previously described to share overlapping clinical and histopathological features, including Castleman disease, Rosai–Dorfman disease, X-linked lymphoproliferative disease, Dianzani autoimmune lymphoproliferative disease, Kikuchi–Fujimoto disease, caspase 8 deficiency syndrome, MAGT1-mediated X-linked immunodeficiency with Mg2 + defect, Epstein–Barr virus infection, and neoplasia disease (XMEN), APDS, CTLA-4 haploinsufficiency with autoimmune infiltration, lipopolysaccharide-responsive vesicle trafficking, and beach- and anchor-containing (LRBA) deficiencies. The molecular mechanism and the physiological purpose of interaction between FAS mutation and sirolimus application remain to be determined in the future analyses. It is possible that this pathway serves to shut down immune reactions to avoid excessive responses and/or limit autoimmune reactions, especially in ALPS-FAS.

## Conclusion

We described a novel germline FAS mutation (c.857G > A, p.G286E) associated with a severe clinical phenotype of ALPS-FAS. Sirolimus effectively improved the patient clinical manifestations with obviously reduction of DNT ratio.

The “Take-away” lesson of this case report is that incidence of ALPS-FAS is extremely low and can easily be confused with the immune-related cytopenia caused by other reasons. Accurate testing of DNT and suspicious genes and related functions is essential for the identification of ALPS-FAS.

## Data availability statement

The datasets for this article are not publicly available due to concerns regarding participant/patient anonymity. Requests to access the datasets should be directed to the corresponding authors.

## Ethics statement

The studies involving human participants were reviewed and approved by the Ethics Committee of Beijing Children’s Hospital, Capital Medical University (January 2, 2021/No. [2021]-E-011-Y). Written informed consent to participate in this study was provided by the participants’ legal guardian. Written informed consent was obtained from the minor(s)’ legal guardian for the publication of any potentially identifiable images or data included in this article.

## Author contributions

HG, ZC, and JYM were performed the material preparation, data collection, and analysis. HG wrote the first draft of the manuscript. All authors commented on the previous versions of the manuscript, contributed to the study conception and design, and read and approved the final manuscript.
